# Tuberculosis Treatment Compliance Under Smartphone-Based Video-Observed Therapy Versus Community-Based Directly Observed Therapy: Cluster Randomized Controlled Trial

**DOI:** 10.2196/53411

**Published:** 2024-06-03

**Authors:** Ponlagrit Kumwichar, Tagoon Prappre, Virasakdi Chongsuvivatwong

**Affiliations:** 1 Department of Epidemiology, Faculty of Medicine, Prince of Songkla University Hat Yai Thailand

**Keywords:** video-enhanced therapy, tuberculosis, health care system, observed therapy, treatment compliance, lung disease, randomized trial, digital health, telehealth, telemedicine, mobile phone

## Abstract

**Background:**

There are no recent studies comparing the compliance rates of both patients and observers in tuberculosis treatment between the video-observed therapy (VOT) and directly observed therapy (DOT) programs.

**Objective:**

This study aims to compare the average number of days that patients with pulmonary tuberculosis and their observers were compliant under VOT and DOT. In addition, this study aims to compare the sputum conversion rate of patients under VOT with that of patients under DOT.

**Methods:**

Patient and observer compliance with tuberculosis treatment between the VOT and DOT programs were compared based on the average number of VOT and DOT compliance days and sputum conversion rates in a 60-day cluster randomized controlled trial with patients with pulmonary tuberculosis (VOT: n=63 and DOT: n=65) with positive sputum acid-fast bacilli smears and 38 observers equally randomized into the VOT and DOT groups (19 observers per group and n=1-5 patients per observer). The VOT group submitted videos to observers via smartphones; the DOT group followed standard procedures. An intention-to-treat analysis assessed the compliance of both the patients and the observers.

**Results:**

The VOT group had higher average compliance than the DOT group (patients: mean difference 15.2 days, 95% CI 4.8-25.6; *P*=.005 and observers: mean difference 21.2 days, 95% CI 13.5-28.9; *P*<.001). The sputum conversion rates in the VOT and DOT groups were 73% and 61.5%, respectively (*P*=.17).

**Conclusions:**

Smartphone-based VOT significantly outperformed community-based DOT in ensuring compliance with tuberculosis treatment among observers. However, the study was underpowered to confirm improved compliance among patients with pulmonary tuberculosis and to detect differences in sputum conversion rates.

**Trial Registration:**

Thai Clinical Trials Registry (TCTR) TCTR20210624002; https://tinyurl.com/3bc2ycrh

**International Registered Report Identifier (IRRID):**

RR2-10.2196/38796

## Introduction

Video-observed therapy (VOT) facilitates remote monitoring of patients with tuberculosis [1] and constitutes an alternative program to directly observed therapy (DOT) [2]. VOT has 2 forms: synchronous VOT (S-VOT) and asynchronous VOT (A-VOT) [1]. In S-VOT, observers video call their patients for real-time observation of drug administration, whereas with A-VOT, observers can review the video sent by the patients at any time. Globally, A-VOT is preferred over S-VOT because it allows patients the flexibility to record drug administration sessions, and the video can be reviewed multiple times [3].

In Thailand, approximately 80,000 tuberculosis cases are reported annually [4]. Since 1996, the country has implemented DOT to ensure treatment adherence [5]. Despite evidence from 2 previous studies indicating the poor sustainability of DOT, no changes have been made due to the lack of alternative strategies and resources [6,7]. The National Tuberculosis Control Program Guideline recommends community-based DOT, observed by health personnel, as the preferred approach [8]. However, in 60% to 75% of tuberculosis cases, family-based DOT is used instead of health personnel observation, reflecting the complacency of the health care system [9,10]. Since 2015, VOT has been used in some areas, without, however, using an accountability system [11]. This system included irregular S-VOT using the LINE (Line Corporation) app or an offline A-VOT that could not be audited daily [11]. The Thai VOT (TH VOT) system, an A-VOT system, has been devised and implemented in Songkhla province, serving as a testing area for the A-VOT system [12,13]. Rather than visiting patient homes in the community as in traditional DOT or performing irregular VOT as previously done, observers can feasibly use the TH VOT system to reduce their travel expenses, and each VOT session performed can be audited daily [12,13]. The TH VOT system is usable and convenient for patients, especially for those who usually take medication late at night [13]. However, the system’s effectiveness in improving medication adherence compared to the traditional community-based DOT remains unknown.

Prior research in Western countries has shown that A-VOT surpasses DOT in ensuring patient adherence, cost-effectiveness, and overall acceptance [14-19]. These investigations evaluated A-VOT based on observation counts. For comparison, counts under DOT, which follow strict regulations, such as those in the United Kingdom and the United States, were also examined [15,18]. In contrast, the observation counts reported from DOT in Thailand are irregular, owing to a low level of accountability among observers [6,7,12]. Therefore, to evaluate the effectiveness of VOT compared to DOT, it is important to consider compliance from both the patients’ and the observers’ perspectives, unlike what has been evaluated in the prior studies [15-18].

The primary objective of this trial was to compare the average number of days that patients with pulmonary tuberculosis and their observers were compliant under VOT and DOT. This was conducted during the intensive phase of treatment, which lasted 60 days, and followed the published protocol [20]. We assumed that medication adherence depended on the compliance of both patients and observers in both the VOT and DOT programs. The results of this study will help determine whether A-VOT can completely replace conventional DOT in Thailand. This is in line with the “Thailand Operation Plan To End TB (2023 to 2027),” which aims at using innovative technology to control tuberculosis [21].

The secondary objective of this trial was to compare the clinical outcomes between the VOT and DOT groups. The clinical outcomes were sputum conversion and reporting of adverse events. This is useful for the future planning of the A-VOT system and for conducting further studies on a larger scale. The eligibility criteria and outcomes were registered before the commencement of the study (TCTR20210624002) [22].

## Methods

### Study Design

We conducted a cluster randomized controlled trial (RCT) in which an observer was assigned to a cluster of patients with pulmonary tuberculosis living in the same jurisdiction, using either DOT or VOT. The trial was registered in the Thai Clinical Trials Registry (TCTR20210624002). The trial protocol followed the CONSORT (Consolidated Standards of Reporting Trials) 2010 statement [23] and is available online [20].

### Study Setting

In Thailand, individuals diagnosed with tuberculosis receive definitive diagnostic evaluations at a hospital located within the jurisdiction of their place of residence. Subsequently, specialized nurses trained in tuberculosis care (tuberculosis nurse) at each hospital delegate a tuberculosis staff member (DOT observer) to administer DOT services to patients located within the corresponding primary care unit (PCU) that aligns with the pertinent jurisdiction.

This study was conducted in the Hat Yai and Meuang Songkhla districts of Songkhla province, Southern Thailand, where a robust internet network is available. Regarding telecommunications services in Thailand, both AIS and TrueMove H corporations offer 4G and 5G networks with speeds that are well above the minimum requirement of 10 Mbps bandwidth for uploading videos from mobile phones [24]. All tuberculosis staff who served as the observers of this study had worked as DOT observers for at least 2 years. All participants were regular smartphone users.

### Background of the Existing A-VOT in Thailand

In Thailand, the TH VOT mobile web system was developed for remote monitoring of antituberculosis drug adherence [12]. This system is accessible through any mobile web browser and is available as an app on Google Play Store [25]. It uses user authentication via the widely used LINE app, with daily LINE notifications for setting the times agreed upon [26-28]. Patients upload videos of their medication intake to the server, which then alerts observers to review these videos. Our previous study, conducted in November 2021, found high patient compliance (approximately 70%) and moderate observer compliance (approximately 50%-65%), despite the challenges posed by the Delta variant of the novel SARS-CoV-2 [13,29]. The system, requiring approximately 1 minute for patient video recording and 1.5 minutes for observer review, proved effective and faced no technical issues in areas with robust internet. It was particularly beneficial for patients taking medication late at night, allowing observers to review videos the following morning [13]. More details on system functions and usability are available on the internet in our previous studies [12,13,20].

### Sampling Method and Recruitment Procedures

In our study area, we randomly selected 53 PCUs using a computer-generated list of random invitations. From these, we invited 38 observers from 38 PCUs based on the list of invitations, leaving 15 PCUs uninvolved. The randomized allocation lists, used for assigning PCUs at a 1:1 ratio to either the VOT or DOT group, were also generated by a computer. Patients with tuberculosis under the jurisdiction of the selected 38 PCUs were assessed for eligibility and subsequently invited to provide informed consent to participate in the study.

### Participants

#### Observers (Cluster Level)

All 38 observers from randomly selected 38 PCUs consented to participate in the study; 19 observers were allocated to the VOT group, and the remaining 19 observers were allocated to the DOT group.

#### Patients (Individual Level)

Patients were considered eligible if they had newly active pulmonary tuberculosis with a positive acid-fast bacilli (AFB) sputum smear, were aged >18 years, owned a smartphone, could use the LINE app, and resided in the same jurisdiction as the observer. Participants were excluded if they had a condition that required specialist intervention, which precluded the 60-day follow-up in intensive phase, rifampicin-resistant tuberculosis evaluated by a cartridge-based nucleic acid amplification test (Xpert MTB and RIF, Cepheid), were unable to continue the treatment for 60 days, or had alcohol dependence.

### Cointerventions

The patients were provided zipped bags daily for 60 days, each with a daily dose of their HRZE (isoniazid, rifampicin, pyrazinamide, and ethambutol) drug regimen [12]. Patients whose consent was registered in the database by a tuberculosis nurse were scheduled to take their medication (HRZE regimen) once daily. After each patient registration, the observer in the jurisdiction where the patient resided was notified through an autonotification of the official LINE (either DOT or VOT).

For monetary compensation, the patients received 300 baht (US $8.68) immediately after registration to cover the cellular internet cost for the first month. Further compensation was paid once the patients completed their 60-day intensive treatment without discontinuing the assigned intervention. They received 300 baht (US $8.68) as a reimbursement for cellular internet cost in the second month and 400 baht (US $11.57) for transportation of the sputum specimen on 3 consecutive days.

The observers who observed medication administration among patients for at least 15 daily sessions out of 60 sessions were compensated with 600 baht (US $17.36). They were also compensated for the cost of travel to visit their patients (4 baht [US $0.12] per km).

### Assigned Interventions

#### Cluster Level

##### VOT for Observers

To avoid a learning curve on the VOT side, the observers performed real or simulated activities for 1 month before the trial [13].

After being notified of patient recruitment, the observers visited the patients at home on the first day. The observer would instruct the patient to redemonstrate the learned procedures [12] as a means of verifying their correct understanding of how to record and upload the video. This training and validation process for independent execution typically required approximately 30 minutes. The observer and patient set a time range for taking the medication, after which the system would send reminder notifications to both the patient and the observer via LINE. Next, the patient maintained a daily record of the drug-taking session, noted any adverse events, and sent a video to the observer through the TH VOT system. The observer reviewed the video, approved the session, and provided necessary advice through the LINE chat box. The observer followed up with a phone call if the patient failed to send the video within 30 minutes of the appointment. If the observers detected any mistakes performed by the patients, they would conduct a video call via LINE to correct the process; these video calls would take approximately 15 minutes.

##### DOT for Observers

Each patient and observer received a session booklet (more details in the protocol by Kumwichar et al [20]). After being notified by the automatic system, the observer conducted a home-visit DOT as a routine service. To validate the observers’ recorded information, the patient and observer were requested to take a photo of the most recent page of the booklet and send it to the auditor through the official TH VOT LINE system every weekend. The auditor reviewed and recorded the number of daily compliance sessions in the database. Note that the observer was independently responsible for managing appointment times with their patients. There was no system support for scheduling appointments to mimic a conventional DOT.

#### Individual Level

##### VOT for Patients

After registration, patients in the VOT group were trained by their observer to record and upload a drug-taking video session according to the standard operating procedure [12]. Briefly, the patients had to set their video frame so that their face was clearly visible. All tablets and capsules should also be clearly visible. They then had to click the “record video” button to start video recording, noting that there is a warning below the button to complain of any nonserious adverse events that may have occurred during the video recording before taking the medication. Patients had to then pick up the pills and place them on their tongue. Next, they swallowed the pills using clear water from a (clear) glass, raised their tongue to show the sublingual area, and stuck out their tongue to show the palatal area. After the drug-taking process was completed, they had to click the “end recording” button to upload the video. After uploading the video to the TH VOT system, the patients could watch an instructional video to remind themselves of the serious adverse effects, which, should they experience, they must stop the medication and call the observer immediately.

##### DOT for Patients

For patients in the DOT group, the tuberculosis nurse provided a booklet to record their daily drug intake and whether the intake was observed by the assigned observer. The tuberculosis nurse requested the patients to return the booklet and all zipped bags on the follow-up day to claim compensation. Each weekend, the auditor notified the patients to capture and send a recent booklet page to the official LINE chat, to which the observers did not have access. All daily reports from patients were recorded without verification, treating them as self-administered treatment.

### Procedures for the Auditor to Review Each Video or Picture Session

#### Sessions in the VOT Group

“Day” was used as the time unit for judging compliance, and local times (GMT +7 hours) were recorded. The *morning* began at midnight, and the evening ended at 11:59 PM. However, daily compliance was judged as “achieved within the cut-off time” if the patients took their medication and submitted their videos before 6 AM on the following day. The auditor assessed the daily video sessions for both the patients and the observers based on the protocol [20].

#### Sessions in the DOT Group

The auditor scored daily compliance weekly based on booklet photos sent by patients with tuberculosis and their observers. The patients were considered to have daily compliance as reported (no audit). The auditor would make a phone call to patients with tuberculosis to confirm whether they were observed as reported by their observer and to remind them to safely store the booklet and all zipped bags received from the tuberculosis clinic, as per the protocol [20].

### Follow-Ups

Each patient was scheduled to return to the tuberculosis clinic for follow-up on day 61. One day before the scheduled visit, the tuberculosis nurse reminded the patients in the DOT group to return the booklet and zipped bags. A deep cough specimen was collected early in the morning for 3 consecutive days (from days 61 to 63). The sputum specimens were subjected to the AFB test. The patients were requested to notify their physicians about all adverse events that occurred at the start of treatment. Physicians recorded the reported adverse events from history using the electronic health record (EHR) system and suggested appropriate treatment. If a patient missed their follow-up appointment, the responsible tuberculosis nurse contacted them and recorded their reasons in the EHR.

### Data Collection

Data regarding observational activities were recorded in the database, and data regarding clinical outcomes were documented in the EHR system of the participating hospitals. The records were retrieved for analysis at the end of the follow-up period.

### Outcomes

#### Primary Outcomes

The data recorded by the auditor were compiled to understand patient and observer compliance in each arm. For the compliance of individual patients, the daily compliance scores rated by the auditor were summed. The mean number of compliance days was calculated for all patients.

Similarly, for compliance of individual observers, daily compliance scores rated by the auditor were calculated. A higher number of patient doses observed increased the mean number of compliance days for the entire group of observers (VOT or DOT).

#### Secondary Outcomes

The clinical outcomes retrieved from the EHR, the conversion of the AFB smear (3 negative sputum smears), the reporting of adverse events, missing follow-up visits, and death during the 60-day follow-up period were compared between the 2 groups.

The information retrieved from the EHR system was used to compare the reporting of adverse events by observers in the VOT and DOT groups.

### Sample Size

Each jurisdictional area comprised 1000 to 5000 individuals. With an approximate annual tuberculosis incidence of 130 per 100,000 individuals in the Songkhla province [30], the sample size estimate was based on the assumption that each cluster could recruit approximately 1 to 5 (mean 3) patients with tuberculosis within 9 months.

The sample size was calculated using the group RCT calculator [31]. The parameters are shown in the protocol [20]. The required number of clusters for each arm was 19. Thus, the number of patients with tuberculosis in each group was 57 (19×3). Using a sample size inflation factor of 20% to compensate for the uncertainty of the tuberculosis incidence in each jurisdictional area, a sample size of 70 patients with tuberculosis was estimated for each arm.

### Cluster Randomized Allocation

Observers who consented to participate were randomly allocated to either the VOT or DOT groups using a file generated using the R software (R Foundation for Statistical Computing). The sequences were stored on a study server. Following the trial protocol, the participating observers registered themselves in the LINE system. After they pressed the “accept” button, the observers were informed about their allocated intervention group through the study LINE system.

### Implementation of the Trial and Patient Information

The new patients with pulmonary tuberculosis were recruited to the VOT or DOT group by a tuberculosis nurse, depending on the jurisdiction of the observer’s residence. Relevant information regarding the study was provided to the potential patients before the start of the trial, including highlighting who could observe them taking medication (their observer and auditor) along with possible assigned interventions (VOT or DOT). The observers’ intervention group was excluded before they consented to participate. If the patients consented to participate, they were assigned to the same intervention group as the observer in their jurisdiction. The participants were free to refuse the intervention at any point after receiving instructions from the tuberculosis nurse. Those who refused to participate or withdrew from the study continued the traditional DOT without data collection compliance. However, clinical data were collected as permitted in accordance with the Thai Personal Data Protection Act, 2019.

### Blinding

The observers disclosed their assigned interventions to auditors, tuberculosis nurses, and researchers. Next, the researchers trained the VOT observers to familiarize themselves with the TH VOT system [13]; the DOT observers were requested to perform traditional DOT as routine care. Therefore, none of the researchers or staff involved in the study were blinded to the assigned interventions.

### Statistical Analysis

Patient and observer background information was summarized using descriptive statistics. An intention-to-treat analysis was conducted according to a randomized allocation. Thus, the participants were classified according to the intervention group to which they were assigned, regardless of whether they changed observation modality. We compared the mean number of compliance days between the 2 groups 60 days after treatment initiation. For a straightforward discussion, we also calculated the compliance rate (%) of each group using the following formula: sum of compliant days for each group×100/(number of patients in each group×60)

Our study was a cluster RCT; thus, the number of compliance days of patients and observers was nested in clusters. We analyzed the 60-day compliance, considering that the same observer may monitor >1 patient. The intervention effect was based on a linear mixed effects model [32]. According to our study design, the intervention was a fixed effect, whereas the cluster level was a random effect. The estimated mean numbers of compliance days along with their SEs for 4 groups of patients under VOT and those under DOT were derived from the model that accounted for the cluster effect.

The number of compliance days for each individual, adjusted for clustering effects, would be calculated based on the specified model. Subsequently, a quantile-quantile (Q-Q) plot would assess the normality of the estimated numbers for each group. If data points predominantly align with a reference line, suggesting normality, a 2-tailed *t* test would be justified for comparing the estimated numbers under VOT versus DOT. Before conducting the *t* test, the Breusch-Pagan test would evaluate homoscedasticity [33]; a *P* value <.05 indicating heteroscedasticity necessitates the use of the Welch *t* test, whereas homoscedastic conditions would permit the application of the Student *t* test. In cases where data markedly deviate from the reference line, indicating non-normality, nonparametric methods would be applied. Data visualization for this study was conducted as outlined in the protocol [20].

Only descriptive statistical methods were used for the secondary outcomes because we did not have sufficient statistical power to detect small differences. The chi-square test was used for comparison; nevertheless, when the expected counts were <5, the Fisher exact test was used.

We also conducted a power analysis using the methodology proposed by Rutterford et al [34] to assess the robustness of our findings. For this analysis, the mean number of patients per cluster was used, and an estimated intraclass correlation coefficient of 0.2 was used to calculate the statistical power, expressed as a percentage. Power calculations were not performed for rare outcomes in which no events occurred in either the DOT or VOT groups.

All analyses were performed using the *epiDisplay* (version 3.5.0.2) [35], *tidyverse* (version 1.3.1) [36], *lmerTest* (version 3.1.3) [37], and *car* (version 3.1-2) [38] packages in R language and environment (version 4.1.1; R Core Team). Statistical significance was established at a 2-sided *P* value of <.05.

### Deviation From the Registered Protocol

In this study, we added calculations for the compliance rate to provide more detail and readability than provided in the statistical analysis outlined in the registered protocol.

### Ethical Considerations

The Human Research Ethics Committee of the Faculty of Medicine, Prince of the Songkla University, approved the trial on February 19, 2021 (REC 64-036-18-9). All participants consented to participate in the trial, allowed access to their data in the EHRs for this research, and consented to the reporting of results in a format in which individuals cannot be identified.

## Results

### Participants

**Figure 1:**
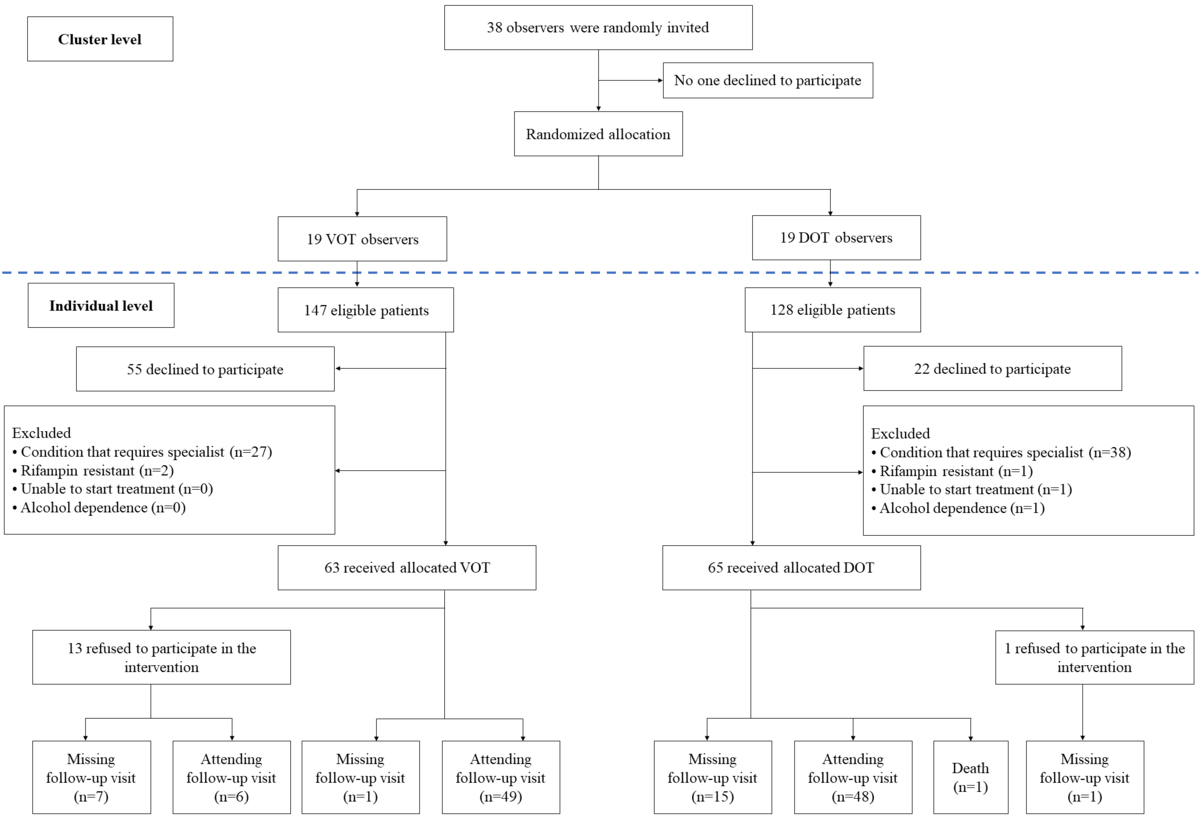
Study flow. Condition that requires specialist: all patients required hospitalization and were transferred to the internal medicine department; follow-up visit: sputum collection and clinical evaluation by a physician. DOT: directly observed therapy; VOT: video-observed therapy.

Between January 2022 and May 2023, a total of 38 observers from 38 PCUs participated in cluster randomization, with 19 (50%) assigned to the VOT group and 19 (50%) to the control group. The trial ended in July 2023 because of a limited budget. A flow diagram of the observers and patients is shown in Figure 1. Eventually, 62.6% (92/147) of the eligible patients in the VOT group and 82.8% (106/128) of the patients in the DOT group consented. Exclusion of patients who consented occurred mainly because they required hospitalization and were transferred to the internal medicine department, which precluded participation in the 60-day intensive phase of VOT or DOT. Of the rest, 63 and 65 patients were recruited in the VOT and DOT groups, respectively. None of the patients changed the modality of observation. However, 21% (13/63) of the patients in the VOT group refused to record videos because of miscommunication with the observers and lack of training with respect to video recording. In the DOT group, 2% (1/65) of the patients refused the intervention after receiving a tutorial on the procedure because of personal concerns. One patient died of severe superimposed pneumonia.

Finally, 87% (55/63) of the individuals in the VOT group returned for a follow-up visit, compared with 74% (48/65) of the individuals in the DOT group. The number of missing follow-up cases was not significantly different between the 2 groups.

All patients assigned to the allocations (63 in the VOT group and 65 in the DOT group) were followed up until the end of the trial. Data from all patients, including those who refused the intervention or died, were analyzed using an intention-to-treat approach.

Table 1 compares the baseline characteristics of the observers and patients in the VOT and DOT groups. The observers in both groups supervised a median of 4 patients each. None of the characteristics showed significant differences in distribution.

**Table 1:** Baseline characteristics of the participants.

Characteristics				VOT^a^		DOT^b^
**Observers**						
	Total, n			19		19
	Age (y), mean (SD)			37.6 (4.9)		35.6 (7.1)
	**Sex, n (%)**					
		Female	11 (58)		13 (68)	
		Male	8 (42)		6 (32)	
	Number of patients under supervision, median (IQR)			4 (2-4)		4 (3-4)
**Patients with pulmonary tuberculosis**						
	Total, n			63		65
	Age (y), mean (SD)			46.3 (14.2)		51.2 (16)
	**Sex, n (%)**					
		Female	21 (33.3)		17 (26.2)	
		Male	42 (66.7)		48 (73.8)	
	Weight (kg), mean (SD)			54.3 (11.1)		52.6 (8.5)
	Height (cm), mean (SD)			163.2 (10.9)		159.9 (9.3)
	BMI (kg/m^2^), mean (SD)			20.4 (3.6)		20.5 (2.6)
	**Pulmonary lesion, n (%)**					
		Left	35 (55.6)		27 (41.5)	
		Right	17 (27)		20 (30.8)	
		Both	11 (17.5)		18 (27.7)	
	Cavitary lesion, n (%)			26 (41.3)		22 (33.8)
	**Underlying disease, n (%)**					
		Diabetes mellitus	11 (17.5)		15 (23.1)	
		HIV infection	3 (4.8)		4 (6.2)	
		COPD^c^	2 (3.2)		1 (1.5)	
		Any cancer	1 (1.6)		1 (1.5)	
	**Number of tablets and capsules prescribed for daily administration, median (IQR)**					
		Isoniazid	3 (2-3)		3 (2-3)	
		Rifampicin	2 (1-2)		2 (1-2)	
		Pyrazinamide	3 (2-3)		3 (2-3)	
		Ethambutol	2 (2-3)		2 (2-2)	

^a^VOT: video-observed therapy.

^b^DOT: directly observed therapy.

^c^COPD: chronic obstructive pulmonary disease.

### Overall Patient and Observer Compliance

**Figure 2:**
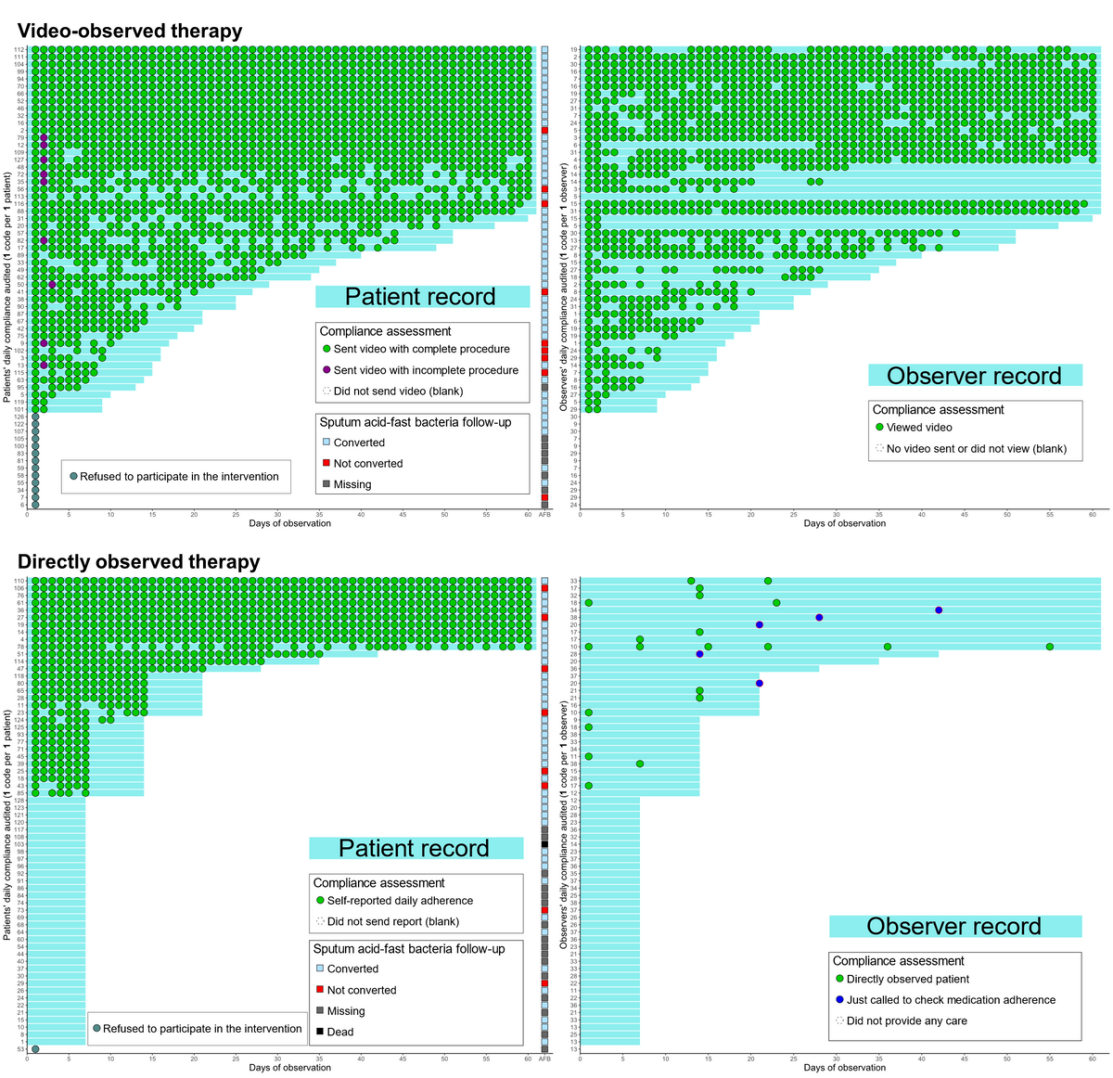
Schematic depicting the 60-day treatment compliance of the patients and their observers in the video-observed therapy and directly observed therapy groups as well as the sputum follow-up results. The blue dots (representing only making a call and not conducting a home visit) were not counted as a compliance day.

Figure 2 shows the 60-day treatment compliance of the patients (left column) and their observers (right column) in the time series of green dots along the x-axis. The y-axis indicates individual records. On the patient side, the last point of the individual time series represents the follow-up AFB smear. Overall, patient compliance correlated with observer compliance. More than half of the patients were unavailable to answer phone calls twice, at which point, they and their observers were discontinued from the compliance assessment.

Within the VOT group, no patient deviated from the recording protocol on day 1 of the compliance assessment because of the oversight provided by their at-home observer. Subsequently, 9 patients failed to comply with the recording protocol but were promptly corrected by their observers. Thereafter, the patients made no further mistakes. Remarkably, without considering the cluster effect, patient compliance rate in the VOT group approached 45.13% (1706/3780) over the 60-day period. In contrast, observer compliance rate was 35.19% (1330/3780).

In contrast, patient compliance rate in the DOT group was 20.9% (815/3900) over the 60-day period. However, the compliance rate of their observers (not including the blue dots in Figure 2) was only 0.54% (21/3900). More than half of the patients (59/65, 91%) initially received no attention. Only 21% (14/65) of the patients were observed at least once. Within 60 days, the number of compliance days for the observers ranged from 0 to 6. Furthermore, some observers simply made a call to check whether their patients had taken their medication without conducting a home visit but reported that the observation was complete (blue dots).

### Outcomes

The cluster-adjusted number of compliance days for each group, as evidenced by the Q-Q plots in Multimedia Appendix 1, is assumed to follow a normal distribution. For VOT versus DOT group comparison, there was evidence of heteroscedasticity by the Breusch-Pagan test as *P*<.001 for patient and observer comparisons. The Welch *t* test was used for comparison of the primary outcomes. Table 2 presents a comparison of the primary and secondary outcomes of the VOT and DOT groups. When comparing the primary outcomes, the average compliance days adjusted for clustering for patients in the VOT group were significantly higher than for those in the DOT group, with a mean difference of 15.2 (95% CI 4.8-25.6). Similarly, VOT observers reported significantly higher average compliance days compared with almost none for DOT observers, with a mean difference of 21.2 (95% CI 13.5-28.9). With the mean number of patients per cluster equal to 3, our study demonstrated sufficient statistical power (>80%) for detecting differences in compliance among observers.

Assessment of the follow-up secondary outcomes showed that 73% (46/63) of the patients in the VOT group achieved sputum AFB smear conversion, compared with 62% (40/65) of the patients in the DOT group (Table 2). The percentage of missed follow-up appointments was notably higher in the DOT cohort, at 25% (16/65), compared with the VOT cohort, at 13% (8/63). The number of adverse events reported by attending physicians was higher than that reported by observers. Overall, in the VOT group, 17% (5/29) of the adverse events recorded by physicians were detected by observers. Conversely, all the adverse events in the DOT group were overlooked by the observers. No statistical significance was detected for comparisons of all secondary outcomes.

**Table 2:** Primary and secondary outcomes evaluated in this study.

Outcomes				VOT^a^ (n=63)		DOT^b^ (n=65)		*P* value		Power (%)
**Primary outcomes**										
	**Estimated mean compliance days per person, mean (SE)^c^**									
		Patients	27.6 (4.4)		12.4 (3.0)		.005		67.5	
		Observers	21.5 (3.5)		0.3 (1.8)		<.001		90.4	
**Secondary outcomes**										
	**Status at follow-up, n (%)**									
		AFB^d^ smear converted^e^	46 (73)		40 (62)		.17		21.3	
		Missed the follow-up visit^e^	8 (13)		16 (25)		.08		30.6	
		Death^f^	0 (0)		1 (1.5)		—^g^		—	
	**Reported adverse events during history taking by a physician, n (%)**									
		Nausea^e^	10 (16)		6 (9)		.26		16.0	
		Rash^e^	9 (14)		3 (5)		.06		35.2	
		Pruritus^f^	5 (8)		4 (6)		.74		5.0	
		Fatigue^f^	3 (5)		1 (2)		.36		14.5	
		Blurred vision^f^	1 (2)		0 (0)		.49		—	
		Numbness	1 (2)		0 (0)		.49		—	
	**Adverse event reported by observers, n (%)**									
		Nausea^f^	3 (5)		0 (0)		.12		—	
		Rash^f^	1 (2)		0 (0)		.49		—	
		Pruritus	0 (0)		0 (0)		—		—	
		Fatigue^f^	1 (2)		0 (0)		.49		—	
		Blurred vision	0 (0)		0 (0)		—		—	
		Numbness	0 (0)		0 (0)		—		—	

^a^VOT: video-observed therapy.

^b^DOT: directly observed therapy.

^c^The mean (SE) was calculated using mixed model linear regression while considering clustering. The Welch *t* test was performed.

^d^AFB: acid-fast bacilli.

^e^Chi-square test was performed.

^f^The Fisher exact test was performed.

^g^Insufficient data for statistical testing.

## Discussion

### Principal Findings

This study assessed medication adherence by evaluating compliance with the experimental intervention in both patients and their observers, using this as a surrogate outcome measure. We assumed that the higher compliance rates among both patients and observers would indicate greater medication adherence in patients. Overall, patients in the VOT group had a notably higher average number of compliance days than those in the DOT group, consistent with the compliance of the observers. However, this study was underpowered to detect improved compliance among the patients. The consent rate in the VOT group (92/147, 62.6%) was lower than that in the DOT group (106/128, 82.8%). This, combined with the fact that 13 patients in the VOT group refused the intervention after consenting due to a lack of support from their observers, implies a need for increased effort from the tuberculosis nurse at the tuberculosis clinic and the observers to enhance patient acceptability of the A-VOT system.

The TH VOT system includes an onscreen reminder before the “record video” button is clicked, prompting patients to report any adverse events in the video before taking their medication. This reminder process was absent in the DOT group. As shown in Table 2, none of the reported adverse events were serious; most patients could endure them. However, without active inquiry about these events by their observers, the patients did not report them. The failure of patients to communicate their adverse events to the observers could be an important factor related to low treatment compliance. Without this care from the observers, patients were less likely to engage in observation therapy, as it would not differ from self-administered therapy (SAT). This issue could be partially mitigated by the reminder interface in the TH VOT system, which prompts users to record a daily video. However, the observers were able to detect only 17% (5/29) of the adverse events retrospectively identified by physicians.

In this RCT, the patient characteristics were well balanced between the VOT and DOT groups. In addition, compliance in both patients and their observers was assessed within clusters, which is more practical than assessing individual effects in a community-based DOT setting [39]. In the VOT group, better compliance was observed and a higher percentage of patients achieved positive AFB smear conversion compared with the DOT group. However, this difference was not significant, potentially due to the limited sample size. The results also suggest a correlation between the compliance of patients and observers. This may indicate the influence of observers on patients in persuading them to adhere to the observation process. Observers in the VOT group reported approximately one-fifth of the adverse events recorded by physicians. In contrast, all these events were completely ignored by the observers in the DOT group. Therefore, training for both VOT and DOT as well as quality control of the observers are of utmost importance.

Previous studies in the United Kingdom and the United States have shown that more than half of the patients received successful observations in the intensive phase at a rate of ≥80% [15,18]. However, our results are less than half of those reported. The possible reasons for this discrepancy include that in Thailand, compulsory observations are required only for patients with extensively drug-resistant tuberculosis [40]. The treatment efficacy of DOT in Thailand has shown no significant difference compared with that of SAT, particularly in nonclinic-based DOT, which often transitions into SAT [5-7]. Given this context, more suitable comparative studies for the findings with the intended-but-failed DOT would have been those exploring the differences in treatment outcomes between VOT and SAT. However, our literature search did not yield any published studies presenting this comparison. Consequently, we referred to data from the United States, indicating that DOT, compared with SAT, resulted in a 40% increase in complete treatment (estimated as odds ratio−1) for individuals with latent tuberculosis infection [41]. Assuming that VOT would be as effective as DOT (as VOT in the United States has been shown to be equivalent to DOT [18,19]), we anticipated that VOT would similarly result in a 40% improvement in complete treatment over SAT in the US context. For our 2-month follow-up, which evaluated sputum conversion rates as a surrogate outcome for complete treatment, we hypothesized that these outcomes would parallel those in the complete treatment observed in the US study. However, the TH VOT system demonstrated only an 18.7% improvement in successful treatment (calculated as [73−61.5]/61.5×100). Compared with those in the United States, this finding underscores the need for more concerted efforts and regulation to enhance treatment success rates in Thailand.

In addition, this study was conducted during the COVID-19 pandemic. Observers might have used the pandemic as a pretext for poor compliance on observation [29]. However, the COVID-19 pandemic was not the main cause of poor compliance among observers, as we noted the poor compliance in our pilot study even before the pandemic began [12]. In addition, there has been evidence of poor compliance with DOT services among observers for >20 years [6,7].

### Limitations

The main limitation of this study was that the trial period was restricted to the first 2 months of tuberculosis treatment (intensive phase). However, although sputum conversion is an uncertain surrogate for successful treatment, many studies have shown that this rate correlates well with treatment success [42-46]. Moreover, blinding was not possible. Nevertheless, both groups were monitored by the same auditor; consequently, the Hawthorne effect should be balanced [47]. In addition, we could not differentiate between the daily doses that were not observed and those that were not taken. Finally, we inferred that the higher compliance of both patients and observers with the assigned intervention indicated better medication adherence. The difference in sputum conversion rate and reporting of adverse events should be interpreted with caution due to inadequate sample size.

The compliance of patients in the VOT group was directly recorded on video to ensure accuracy. In contrast, compliance in the DOT group was based solely on patient reports, which could not be verified. The statistics based on potential overreporting in the DOT group may have biased our results toward the underestimation of the superiority of the A-VOT system over DOT. This disparity in compliance quality limited the comparison of the 2 groups. Even so, the reported compliance in the DOT group may have been overestimated; however, it was still lower than that in the VOT group. This might be because the VOT system is more feasible than the traditional DOT and its notification system can enhance observer compliance [13]. Consequently, it may indirectly improve patient compliance through increased encouragement and response from observers [48].

Staff time and effort to train and supervise drug intake should be considered during the implementation of the A-VOT system. The A-VOT could reduce the travel time of the observers substantially. On the contrary, effort to train patients to use the A-VOT system, especially patients who are from a low socioeconomic background, and session recording supervision by S-VOT may be necessary for the first few days, when patients are still unfamiliar with the system.

### Conclusions

In Thailand, although A-VOT requires more initial effort and has lower acceptability, it was superior to traditional community-based DOT in ensuring treatment compliance among observers. Nonetheless, the study lacked the statistical power to validate enhanced adherence to treatment among patients with pulmonary tuberculosis and to detect differences in sputum conversion rates. In community-based DOT settings with robust internet availability, replacing the DOT program with the A-VOT system may improve medication adherence among patients with tuberculosis, although a more accountable system for the observers is needed.

## Appendix

Multimedia Appendix 1 Supplementary figures.

Multimedia Appendix 2 CONSORT-eHEALTH checklist (V 1.6.1).

## Abbreviations

AFB: acid-fast bacilli
A-VOT: asynchronous video-observed therapy
CONSORT: Consolidated Standards of Reporting Trials
DOT: directly observed therapy
EHR: electronic health record
HRZE: isoniazid, rifampicin, pyrazinamide, and ethambutol
PCU: primary care unit
RCT: randomized controlled trial
SAT: self-administered therapy
S-VOT: synchronous video-observed therapy
TH VOT: Thai video-observed therapy
VOT: video-observed therapy

## Data Availability

The data used in this study were deidentified to ensure patient confidentiality in accordance with the Personal Data Protection Act BE 2562 of Thailand. Therefore, the data could not be used to indirectly identify a person; however, they were sufficiently detailed to enable analysis and yield results consistent with the original data. The research data have been shared on GitHub [49].
