# Supplementary material for: Tuberculosis Treatment Compliance Under Smartphone-Based Video-Observed Therapy Versus Community-Based Directly Observed Therapy: Cluster Randomized Controlled Trial
Source: JMIR Mhealth Uhealth. 2024 Jun 3;12:e53411. doi: 10.2196/53411 (PMC11184266; doi:10.2196/53411)
Supplement: Multimedia Appendix 1 [file mhealth_v12i1e53411_app1.docx]

# **Supplementary data**

# Figures

#
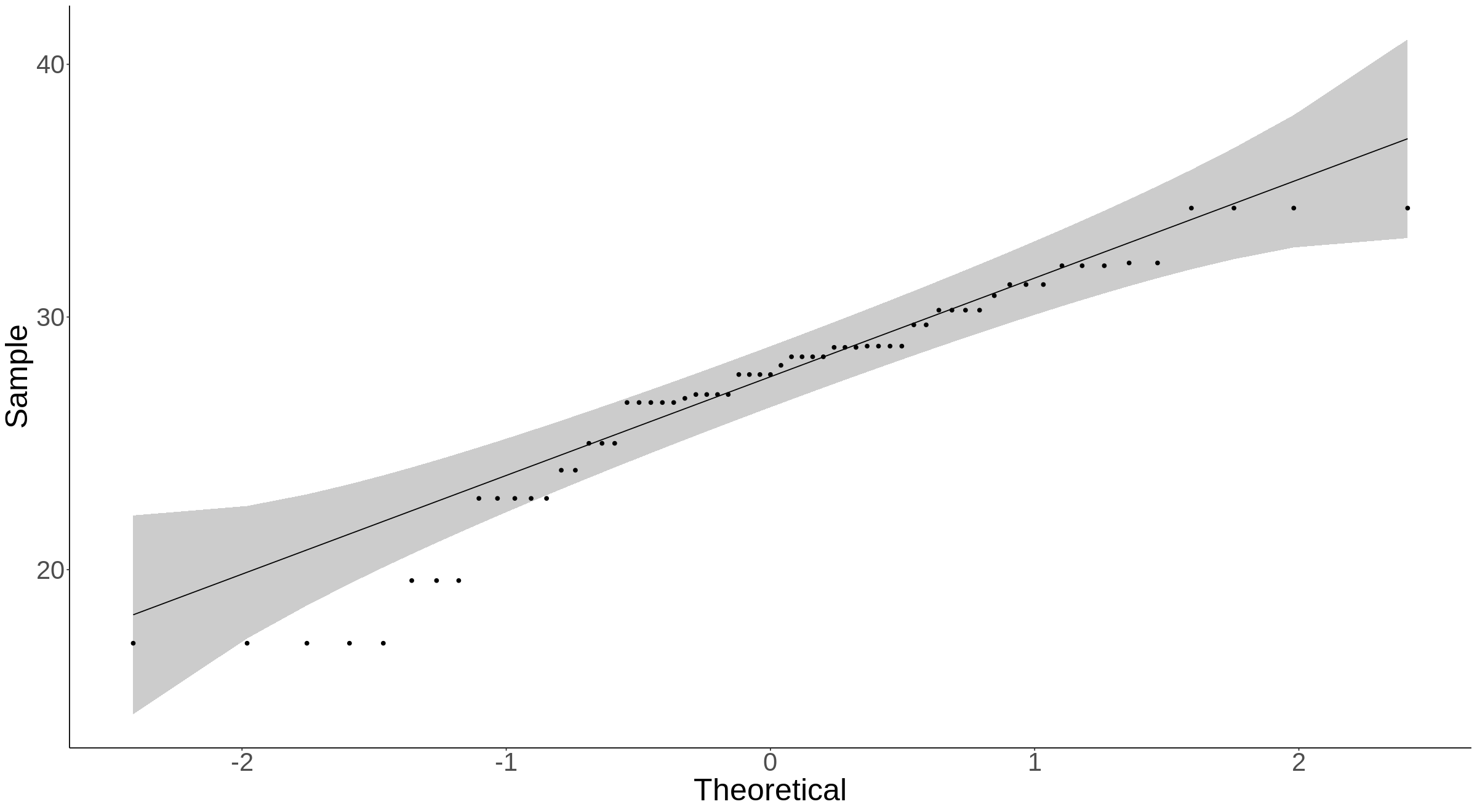


**Figure S1.** Quantile-Quantile plot comparing the cluster-adjusted number of compliance days for patients under video observed therapy to a theoretical normal distribution.


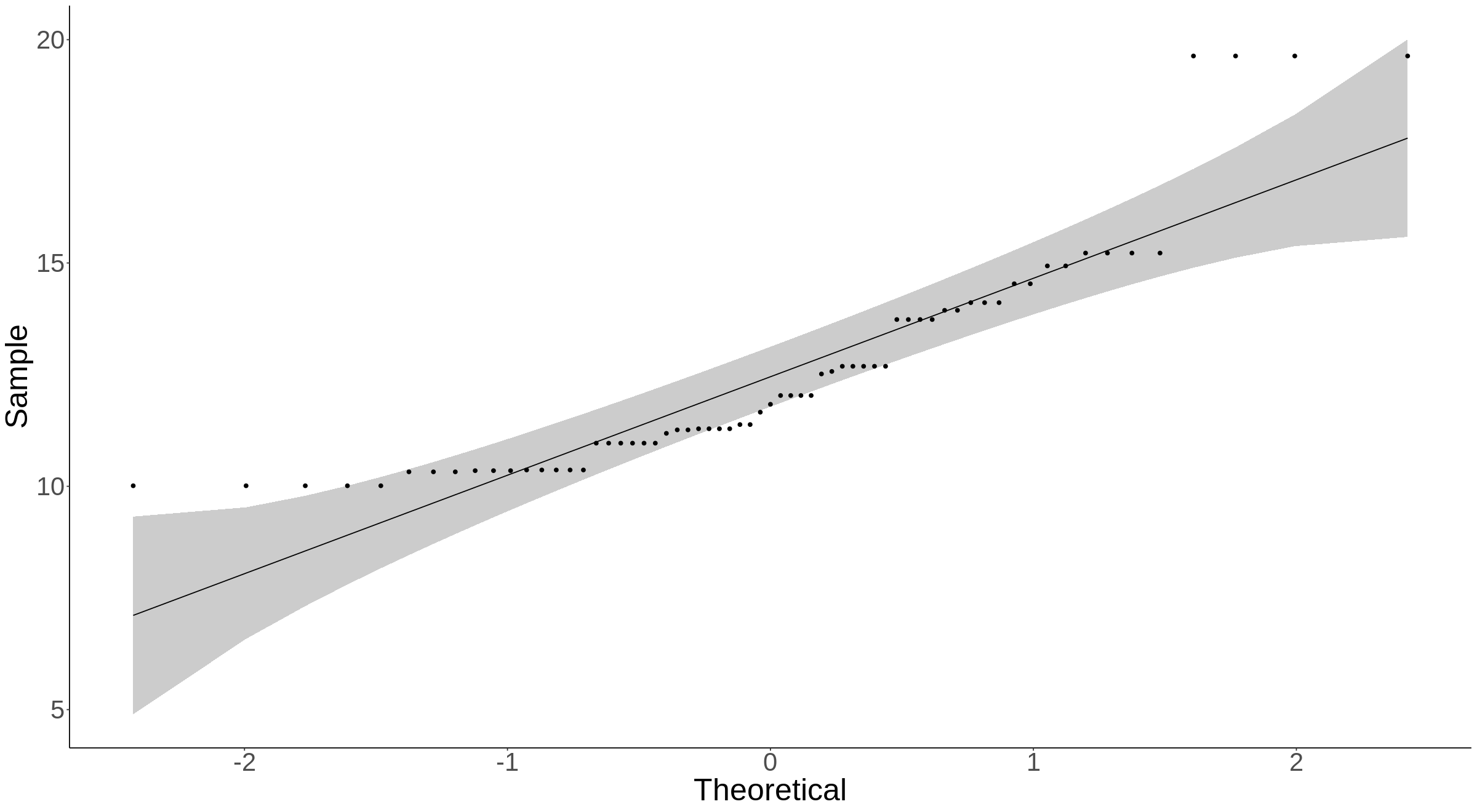


**Figure S2.** Quantile-Quantile plot comparing the cluster-adjusted number of compliance days for patients under directly observed therapy to a theoretical normal distribution.


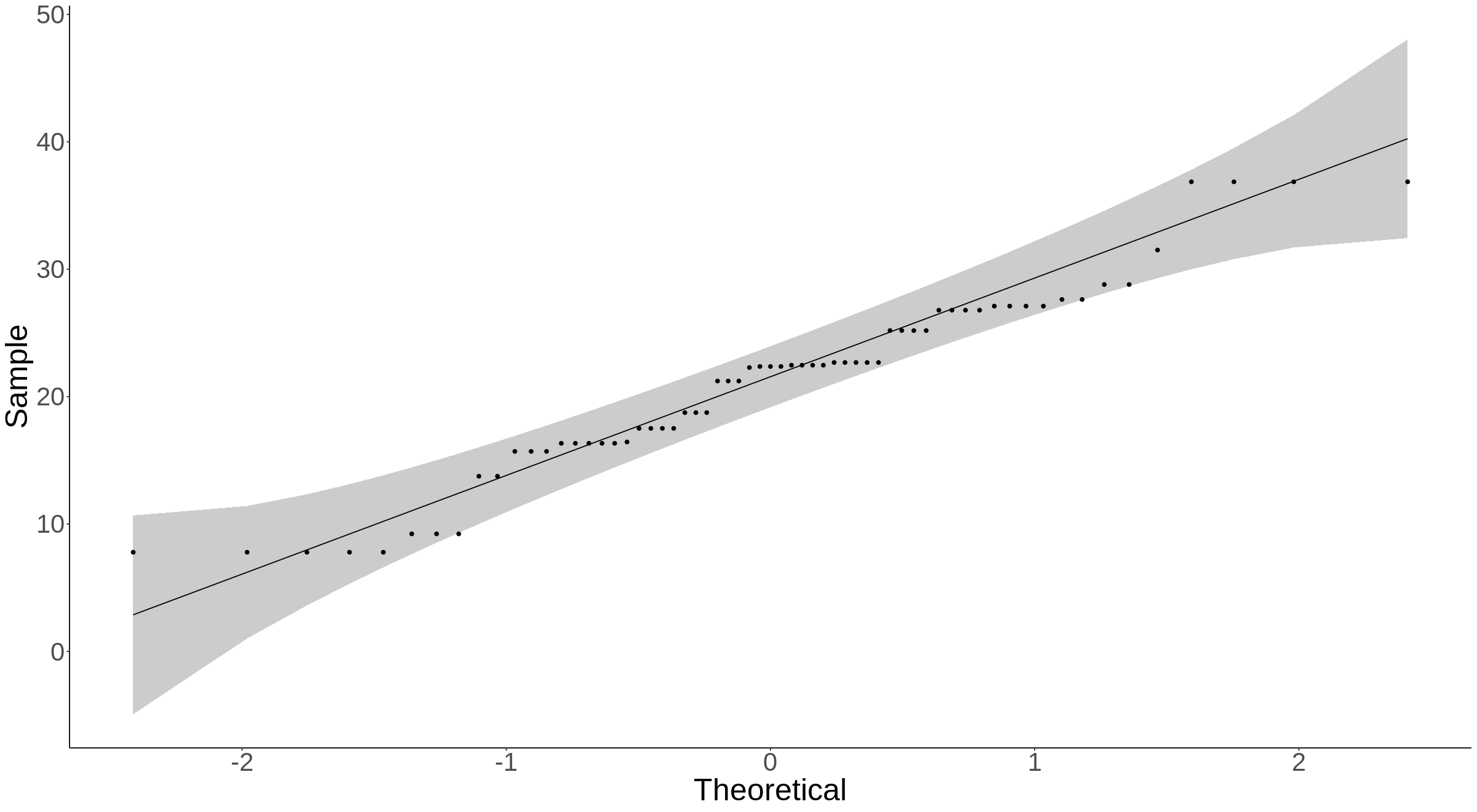


**Figure S3.** Quantile-Quantile plot comparing the cluster-adjusted number of compliance days for observers providing video observed therapy to a theoretical normal distribution.


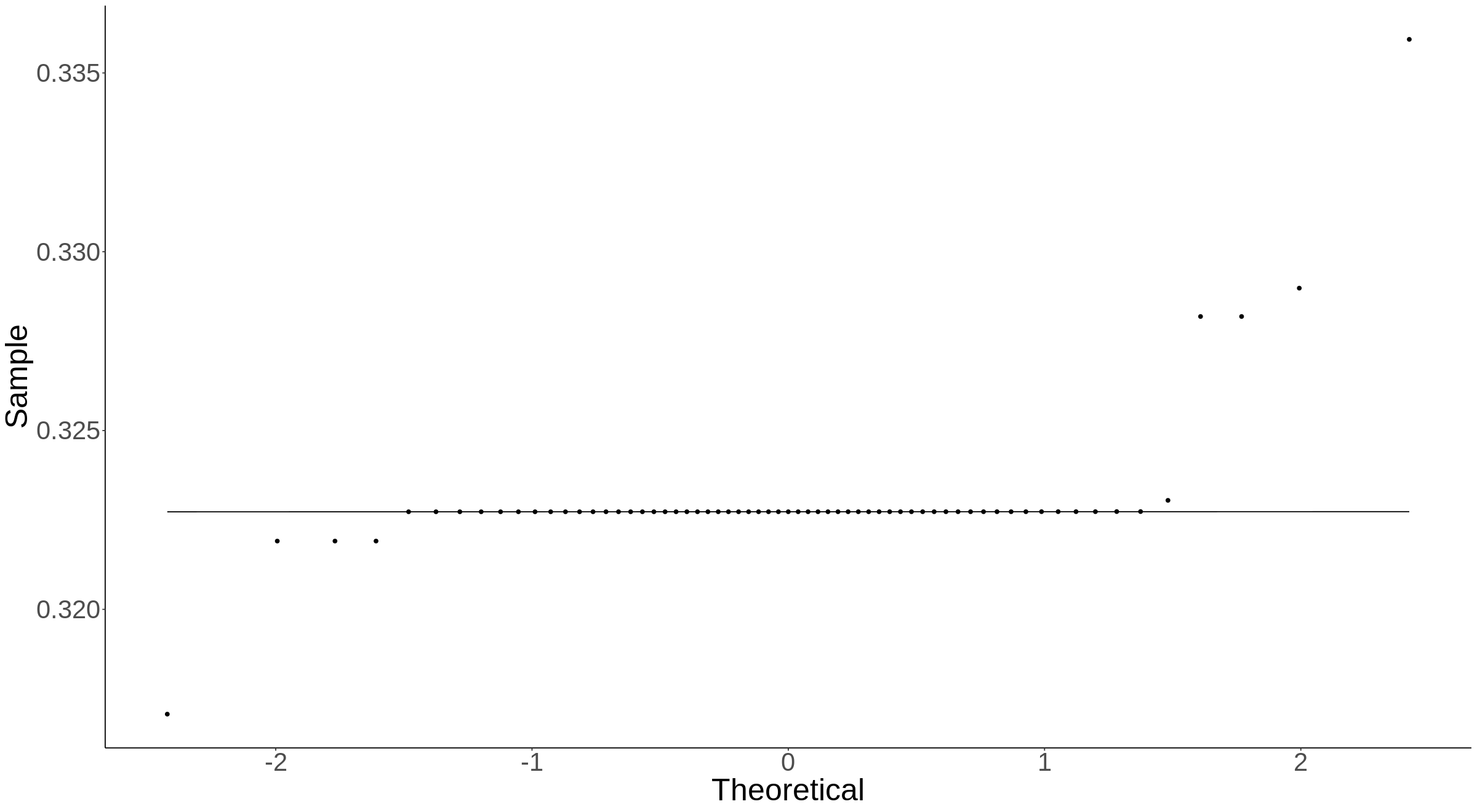


**Figure S4.** Quantile-Quantile plot comparing the cluster-adjusted number of compliance days for observers providing directly observed therapy to a theoretical normal distribution.
